# A Review on Hydrogels with Photothermal Effect in Wound Healing and Bone Tissue Engineering

**DOI:** 10.3390/polym13132100

**Published:** 2021-06-25

**Authors:** Xu Zhang, Bowen Tan, Yanting Wu, Min Zhang, Jinfeng Liao

**Affiliations:** State Key Laboratory of Oral Diseases, National Clinical Research Centre for Oral Diseases, West China Hospital of Stomatology, Sichuan University, Chengdu 610041, China; zx2020224030006@163.com (X.Z.); bowentan711@163.com (B.T.); yantingwu0521@outlook.com (Y.W.); zhangmin5477@163.com (M.Z.)

**Keywords:** hydrogel, photothermal effect, antibacterial, angiogenesis, wound healing, bone regeneration

## Abstract

Photothermal treatment (PTT) is a promising strategy to deal with multidrug-resistant bacteria infection and promote tissue regeneration. Previous studies demonstrated that hyperthermia can effectively inhibit the growth of bacteria, whereas mild heat can promote cell proliferation, further accelerating wound healing and bone regeneration. Especially, hydrogels with photothermal properties could achieve remotely controlled drug release. In this review, we introduce a photothermal agent hybrid in hydrogels for a photothermal effect. We also summarize the potential mechanisms of photothermal hydrogels regarding antibacterial action, angiogenesis, and osteogenesis. Furthermore, recent developments in photothermal hydrogels in wound healing and bone regeneration applications are introduced. Finally, future application of photothermal hydrogels is discussed. Hydrogels with photothermal effects provide a new direction for wound healing and bone regeneration, and this review will give a reference for the tissue engineering.

## 1. Introduction

Tumors [1], fractures [2], burns [3], diabetes [4], and other acute/chronic diseases seriously affect people’s health. These diseases are accompanied by severe infections or tissue defects. In clinical practice, doctors use large doses of antibiotics to control infection and artificial substitutes to repair tissue defects. However, this may lead to the emergence of drug-resistant bacteria and tissue inflammation around implants. With the rapid development of bio-nanotechnology, researchers have developed various kinds of biomaterials such as hydrogels [5], films [6], sponges [7], cements [8], microneedles [9], and three-dimensional (3D)-printed scaffolds [10] to solve these problems.

Hydrogels, as a 3D porous material, can keep a moist microenvironment, absorb inflammation exudate, and provide a physical barrier to prevent bacteria from entering the wound [11,12]. Other features of hydrogels include their excellent biocompatibility, adjustable degradation properties, and proper mechanical properties [13,14]. Researchers have developed different kinds of hydrogels that are suitable for different defect sites. Injectable hydrogels can complete sol-gel conversion in a short time via hydrogen bonds [15,16], host–guest interactions [17], dynamic Schiff bonds [18], dynamic diol-borate ester crosslinking [19], and the Diels−Alder reaction [20]. Hence, injectable hydrogels can be applied to irregular defect areas. However, external shear stress may destroy the internal structure of hydrogels, and even lead to gel-sol conversion. Hydrogels polymerized from monomers have excellent stability and high mechanical strength. The cross-linked hydrogels are synthesized mainly by polymerization with carbon–carbon double bonds. The monomers used commonly are acrylamide [21], N-isopropylacrylamide [22,23], and gelatin methacrylate (GelMA) [24]. Moreover, ionic cross-linking hydrogels, such as Ca^2+^ cross-linked sodium alginate hydrogel, has attracted much attention because of its convenient cross-linking and stable performance [25,26]. Hydrogels mimic the extracellular matrix to promote cell proliferation and further accelerate tissue regeneration [27].

Several recent studies have focused their attention on changes in the external temperature that influence the responses of bacterial, cells, and tissues. Photothermal agents can convert light energy to heat under near infrared (NIR) irradiation [28,29]. The target temperature of photothermal hydrogels can be adjusted by changing the concentration and proportion of photothermal agents, irradiation time, and laser intensity [30]. Mild local heat (41 °C–43 °C) can promote cell proliferation, angiogenesis, wound healing, and bone regeneration [31]. Moderate heat (45 °C–50 °C) causes negligible damage to normal tissue cells in a short time but fatal damage to tumor cells [32,33,34]. For infected wound healing, hyperthermia (>50 °C) can effectively inhibit the proliferation of bacteria. Therefore, photothermal effects can be controlled for different applications based on different temperature.

The change in temperature can control the gel-sol transformation of thermosensitive hydrogels and release active factors for enhanced therapy [35]. Pedersen et al. reported that a polyvinyl alcohol/gold nanorod (AuNR) hydrogel was heated to <50 °C under NIR irradiation and liquefied in 15–20 s, and the pulsed release model cargo of fluorescein isothiocyanate controlled by NIR irradiation was realized successfully [36]. Under NIR irradiation, [poly(d,l-lactide)-poly(ethylene glycol)-poly(d,l-lactide)/black phosphorus (BP) hydrogel undergo a sol-gel transition at 46.5 °C after only 20 s [37]. Hyperthermia (>50 °C) could destroy the integrity of bacterial cell membranes, cause protein denaturation, and finally kill bacteria [38]. Hyperthermia caused by long-term exposure to NIR laser can also result in irreversible damage to normal tissues. To minimize the negative influence on normal tissues, antibacterial substances are usually incorporated into hydrogels to work with photothermal effects. Hydrogels can load drugs via hydrogen bonds [39], π−π stacking [40], dynamic chemical bonds [41], and electrostatic absorption [42,43]. Furthermore, hyperthermia can effectively alleviate intermolecular forces or shrink volume to achieve rapid drug release to enhance the therapeutic effect [44,45,46,47].

Therefore, photothermal hydrogels combine the advantages of traditional hydrogels with the effect of temperature stimulation on tissues. Controlling the final temperature of the hydrogels enables them to be used in different biological treatment strategies. Different types of hydrogels can be selected according to the application sites and therapeutic strategies. In this review, the fabrication and characteristics of photothermal hydrogels are introduced. Photothermal hydrogels showed good applications in wound healing and bone regeneration in recent years. Furthermore, the future perspective of photothermal hydrogels in the biomedical field is discussed.

## 2. Composition of Hydrogels with Photothermal Effects

Materials with photothermal effects have been applied widely in antitumor treatment, and increasing applications in antibacterial effect, wound healing, and tissue regeneration have been reported in the past 2–3 years [48]. According to the different sources of photothermal initiators, materials with photothermal effects can be divided into two types: inorganic materials and organic materials. Inorganic materials are usually nanomaterials and show strong NIR absorption properties and possess a stable photothermal effect. However, a high concentration of nanomaterials may be toxic to tissue and easy to aggregate. Compared with inorganic materials, organic materials show approving biocompatibility and are readily available. However, organic materials with photothermal effect often have photobleaching effect. In the following sections, hydrogels with different kinds of photothermal agents are introduced systematically.

### 2.1. Inorganic Materials Hybrid Hydrogels with Photothermal Effects

The inorganic materials with photothermal effects include carbon nanotubes [49,50,51], reduced graphene oxide (rGO) [52,53], gold nanoparticles [54,55,56], BP [57,58], AuPds [31], silver [59], TiO_2_ [60], and platinum nanoparticles [61]. However, the aggregation of nanoparticles in organisms limits their applications. Hydrogels with a porous network have been used widely in biomedicine. One of the strategies to solve aggregation is that nanomaterials can be dispersed effectively in hydrogels [62,63]. In addition, nanomaterials can be surface-modified and covalently cross-linked or non-covalently cross-linked with hydrogels [64,65].

Chitosan (CS)/antimonene nanosheets (AMNSs) hydrogels were prepared via a bidirectional freeze-casting approach by Liu’s group. Scanning electron microscope (SEM) images showed that the layered AMNSs were embedded evenly in the framework of the CS hydrogel [66]. GO lacks stability in a physiological cycle. Surface modification could improve its stability and further form a covalent bond with the bulk of the hydrogel. The amino group can be obtained by grafting branched poly-ethylenimine (BPEI) onto graphene oxide, which could be cross-linked with the aldehyde group to form a dynamic Schiff base bond [67]. Some metal–organic frameworks have been developed for photothermal therapy. For example, N, N-bis (acryloyl) cystamine can effectively adsorb copper nanoparticles via Cu–S bonds [68]. Moreover, polyvinyl pyrrolidone (PVP) functionalized GO nanosheets were crosslinked with AuNRs via non-covalent electrochemical interactions. Then PVP-nGO@AuNRs can be added into interpenetrating network (IPN) formed hybrid hydrogel by free radical polymerization [55]. To avoid nanomaterials aggregation, Prussian blue (PB) can be surface modified by PDA to anchor Ag^+^ and achieve controlled release Ag^+^. Moreover, due to high photothermal conversion property and excellent stability of PB, PB has been applied in antibacterial therapy and wound healing [69,70].

### 2.2. Organic Materials Hybrid Hydrogels with Photothermal Effects

Organic materials with photothermal conversion ability have also received extensive attention because of their good degradation properties and excellent biocompatibility. Research studies on organic photothermal conversion materials focused on dyes such as IR-820 [71,72], IR-780 [73,74], indocyanine green (ICG) [75], and conjugated polymers such as poly-dopamine (PDA) [76,77], poly-pyrrole [78,79], and poly-aniline [80].

Dopamine (DA), as a material with excellent photothermal conversion and adhesion abilities, has attracted increasing attention. DA can be grafted onto polymer materials, such as GelMA [81], hyaluronic acid(HA) [82], and bioactive glass nanoparticles (BGNs) [83], to improve its adhesion ability. In addition, DA could chelate with other photothermal materials (such as Cu^2+^ [84], and Ti [85]) to increase the photothermal performance of hydrogels. Furthermore, the catechol group can be easily oxidized to the catechol–quinone group, which further forms chemical cross-links to improve the mechanical properties of hydrogels [86,87]. PDA can be finally formed by oxidative and DA self-polymerization in a basic solution [88]. PDA can load the drug through π−π stacking, hydrogen bonds, and electrostatic attraction. Tong et al. recently fabricated Curdlan powder/PDA hydrogel and successfully loaded chlorhexidine (CHX). CHX is an antibacterial drug, and was used to gain a controllable NIR-triggered release [89]. Moreover, PDA can be incorporated with GO through π−π stacking to regulate the NIR-triggered release and reduce drug leakage [90]. 

ICG [41,91], as an FDA-approved photothermal therapeutic agent, has also attracted increasing attention owing to its remarkable photothermal conversion efficiency. ICG can also improve photothermal therapeutic effects by incorporating rare-earth nanoparticles (Ln: 72% Y^3+^, 20% Yb^3+^,8% Tm^3+^) via electrostatic adsorption. Li et al. recently created an ICG–rare-earth nanoparticle-loaded hydrogel, with the successful implementation of NIR-triggered release of adriamycin (DOX) [92].

## 3. Potential Mechanisms of Photothermal Hydrogels for Wound Healing and Tissue Engineering

To achieve the flexible use of photothermal hydrogels in wound healing and bone tissue engineering, it is necessary to explore the potential mechanisms of photothermal effects in antibacterial activity, angiogenesis, and osteogenesis. Mild local heat can improve the expression of various genes that promote tissue repair, thereby promoting cell proliferation and differentiation, and ultimately accelerating wound healing and bone regeneration. In wound healing and bone regeneration, oxygen and nutrition supply from neovascularization is essential. When the wound is seriously infected, antibodies in the blood can control the infection. Based on controlling infection and angiogenesis, bone marrow mesenchymal stem cells (BMSCs) can proliferate and further promote tissue regeneration.

### 3.1. Antibacterial Effects

**Figure 1:**
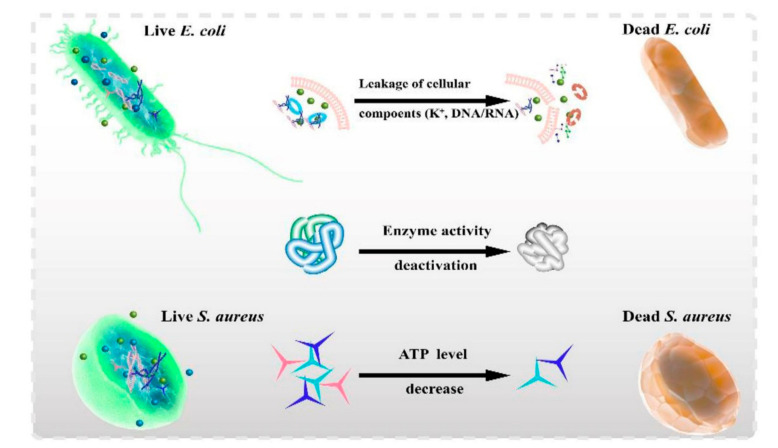
Hyperthermia effects on the antibacteria via changing cell membrane permeability, enzyme inactivation, and protein denaturation [95]. Copyright 2021, Elsevier.

Recent researches disclosed that high temperature (>50 °C) can effectively kill bacteria [93]. Hence, materials with high photothermal conversion efficiency have gradually caught the attention of researchers. High temperature could cause enzyme inactivation, cell membrane permeability changes [66,82], cytoplasmic outflow [66], and protein denaturation [94] (Figure 1). The cell membranes of *Escherichia* *coli* became shrunken and blurred after NIR irradiation, and the integrity of the cell membranes was destroyed, indicating that high temperature can destroy bacterial cell membranes [95]. Moreover, Li et al. demonstrated that the photothermal effect can destroy bacterial cell membranes, denature bacterial proteins, and change cell membrane permeability [96]. Further, the bacterial intracellular matrix flowed out when the integrity of the cell membranes and cell walls was destroyed. Using transmission electron microscopy, Liu et al. found that the bacterial cell membranes were destroyed, resulting in cytoplasm outflow [97].

**Figure 2:**
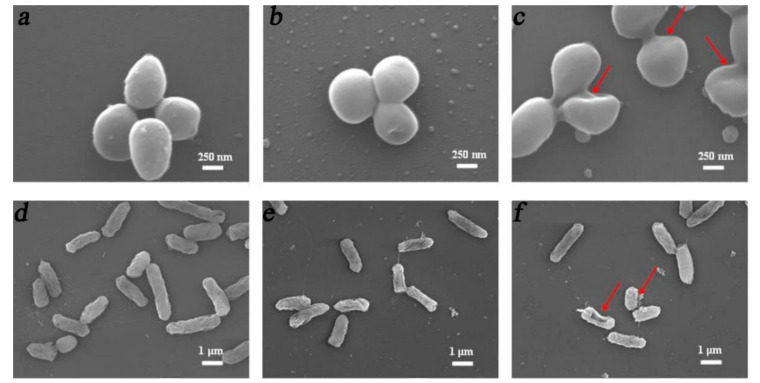
SEM images of S. aureus (**a**–**c**) and *E. coli* (**d**–**f**) after culture on PVA/CuS/MoS_2_ hydrogels with NIR/visual light irradiation for 15 min. (**a**,**d**) PVA hydrogel, (**b**,**e**) CuS incorporated PVA hydrogel, and (**c,f**) CuS/MoS_2_-incorporated PVA hydrogel [104]. Copyright 2020, Elsevier.

In the application of photothermal therapy, hyperthermia recovers to body temperature in a short time. To improve the treatment efficacy, strategies include increasing the irradiation time and adding bioactive agents for co-therapy. Photothermal antibacterial materials can be combined with various antibacterial substances, (such as Cu_2_O [98], Ag^+^ [99,100], and Au [101]), to increase antibacterial effect. The antimicrobial metal ions and metal oxides can act synergistically with photothermal materials to increase the antimicrobial properties of the composite material. A “one stone, two birds” strategy was used, based on the surface plasmon resonance (SPR) effect of Ag^+^ and Cu^2+^ to kill bacteria by photothermal therapy and achieve a sustained and stable release of bioactive ions with antibacterial properties [102,103]. Interestingly, in another study, copper ions were incorporated with NIR irradiation, and bacteria were inhibited quickly and continuously because of the “hot ions effect”. Cyclic NIR irradiation was used to verify the antibacterial properties of the hot ions effect. In the first cycle, the antibacterial activity of a PDA/Cu hydrogel against *E. coli* reached 88.37%, whereas the Cu group showed poor antibacterial activity (9.30%). After the second NIR irradiation cycle of 2 h, the antibacterial rate of the PDA/Cu hydrogel was 98.53%, whereas the antibacterial rate of the hydrogel without NIR irradiation was 79.48% [84]. Moreover, the CuS/MoS_2_/PVA hydrogel could produce hyperthermia and reactive oxygen species under NIR/visual light to provide an antibacterial treatment. As shown in Figure 2, after the bacteria were incubated with the hydrogel, the cell membranes were damaged to varying degrees [104] 

Hydrogels can be incorporated with drugs to improve antibacterial properties via π−π stacking, reversible covalent cross-links [105], and direct encapsulation [106]. A photothermal trigger or pH trigger can be chosen to control drug release [107]. Liu et al. revealed that MPDA/GO/CNF composite hydrogel could be easily loaded with tetracycline hydrochloride (TH) via π−π stacking and hydrogen bonds. When the pH changes from a neutral to an acidic condition, the amino group on the surface of PDA changed from deprotonation to protonation, further disrupting the attachment between TH and PDA. In addition, hyper-temperature could alleviate π−π stacking to achieve a rapid release of TH [90]. Curcumin is an aromatic hydrocarbon material used widely for its anti-inflammatory, antibacterial, and antitumor activities [108]. A gel-PDA/Cur hydrogel could form π−π stacking with curcumin. Under NIR irradiation, curcumin can be released rapidly compared to only a slight release without NIR. This phenomenon can be attributed to the fact that π−π stacking and hydrogen bonds can be destroyed by hyperthermia [95,109]. Doxycycline, as a broad-spectrum antibiotic agent, has been used widely in clinical practice [110]. The amino group on doxycycline can form a hydrogen bond with HA-DA/rGO hydrogel to prolong the drug release time [93]. Yang et al. constructed dodecyl-modified CS/PEG–CHO/Tungsten disulfide nanosheets hydrogel to load ciprofloxacin *via* hydrogen bonding and electrostatic interactions, and NIR-triggered ciprofloxacin release was achieved successfully [111]. 

### 3.2. Angiogenesis

**Figure 3:**
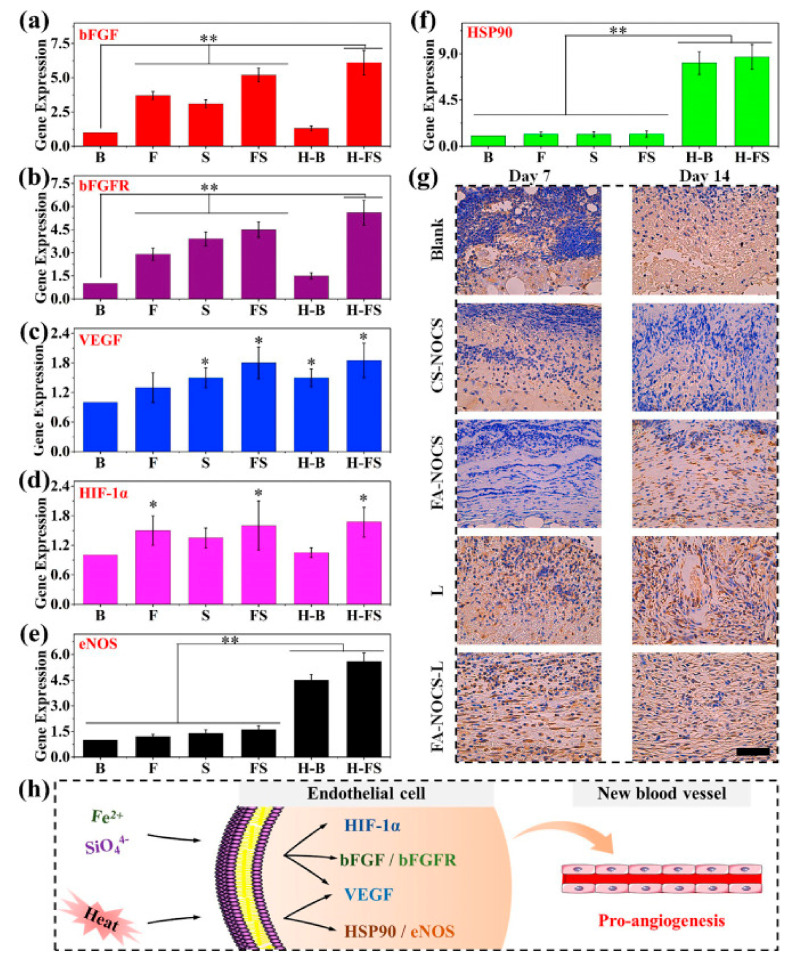
The effect of heat stimulation and Fe/Si ions on gene (in vitro) and protein (in vivo) expression. (**a**) bFGF, (**b**) bFGFR, (**c**) VEGF, (**d**) HIF-1α, (**e**) eNOS, (**f**) HSP90 gene expression of HUVECs at day 7. (**g**) Immunohistochemical staining for HSP90 (Blank: no treatment; CS-NOCS: calcium silicate-NOCS composite hydrogels without NIR irradiation; FA-NOCS: FA-NOCS composite hydrogels without NIR irradiation; FA–NOCS–L: FA-NOCS composite hydrogels with NIR irradiation (808 nm, 0.36 W/cm^2^, 15min/day, day 1–5); L: laser irradiation only; Bar = 50 μm). (**h**) Possible activation mechanism of the combined effect of ions and thermal stimulation on angiogenesis. (B: blank; F: Fe^2+^ containing medium; S: SiO_4_^4−^ containing medium; FS: Fe^2+^ and SiO_4_^4−^ containing medium; H–B: heat treatment; H-FS: combination of heat and Fe^2+^ and SiO_4_^4−^ ion treatment, * *p* < 0.05, ** *p* < 0.01). [112] Copyright 2020, Elsevier.

In chronic wound healing and bone tissue repair, hypoxic cells are usually unable to proliferate normally owing to lack of nutrients. Angiogenesis can provide oxygen and nutrients to the newborn tissue, thus helping to accelerate wound healing and bone repair. Recent studies have found that mild heat (40 °C–41 °C) can effectively increase the vascular density in the new granulation tissue by inducing the proliferation of vascular endothelial cells. Moreover, by simulating the “hot spring effect”, mild heat can be incorporated with Fe^2+^, SiO_4_^4−^ ions to regulate the expression of vascular genes such as hypoxia inducible factor-1 (HIF-1α), vascular endothelial growth factor (VEGF), endothelial nitric oxide synthase (eNOs), basic fibroblast growth factor (bFGF), and further promote circulation of blood (Figure 3) [112]. Moreover, Cu^2+^ and Mo^4+^ ions could promote angiogenesis by promoting the expression of related genes (VEGF, KDR, HIF-α, eNOs, bFGF) [68,84]. HIF-α can mimic hypoxia environment to induce cell differentiation. Some ions, such as Cu^2+^, Mo^4+^, and SiO_4_^4−^, can also promote collagen deposition, hair follicle regeneration, and fibroblast proliferation, and accelerate tissue healing [113,114,115].

Adequate oxygen could promote cell proliferation and tissue regeneration. However, it is difficult for most oxygen carriers to release oxygen into deep tissues [116]. In addition, CO_2_ can stimulate angiogenesis in the wound area via the Bohr effect. The Bohr effect means that the hemoglobin releases more oxygen when the pH of the blood decreases. Bicarbonate decomposes into CO_2_ under mild heat (42 °C). Bicarbonate can be coordinated with Fe^3+^ and bound to the PDA hydrogel network, further achieving the controlled release of CO_2_ triggered by photothermal conversion [117,118]. Moreover, NO, an important signal regulation molecule, could promote angiogenesis via multiple mechanisms [119]. Recent research suggested that excess NO did not show satisfactory results compared to a small quantity of NO, and excessive NO release of NO may inhibit cell proliferation. So, controlled NO release is a key factor in its application. As a material that can continuously release NO under NIR irradiation, the hydrophobic BNN6 limits its application. Hydrogels as a hydrophilic 3D network structure material carrier can encapsulate hydrophobic agents to disperse them well [82,120]. Zeolitic imidazolate framework-8 (ZIF-8), as a novel metal–organic framework, could absorb many small molecules because of its positively charged surfaces and efficiently improve water solubility. Liu et al. recently successfully achieved the NIR-controlled release of NO by encapsulating ZIF-8@BNN6 nanoparticles in a GelMA/oxide dextran hydrogel. With the increase in the number of NIR irradiation cycles, the NO produced by the decomposition of BNN6 exhibited a cumulative effect [121]. 

### 3.3. Osteogenesis

Compared with short photothermal stimulation without obvious effects on osteogenesis, periodic photothermal treatment can stimulate the proliferation and differentiation of BMSCs, which are attributed to the prolongation thermal effect. Mild heat stimulation (41 °C once a week for 1 h) could enhance the expression of alkaline phosphatase (ALP) to promote BMSCs differentiation into osteoblasts [122]. Some direct proofs also showed that weekly photothermal stimulation after 10 weeks can promote bone regeneration efficiently in vivo. Micro-computed tomography images showed that the bone volume/total volume (BV/TV) ratio of the NIR irradiation + poly (lactic-co-glycolic acid) (PLGA) group was 23.50% ± 1.12% and that of the only PLGA group was 12.02% ± 1.30% [57]. Moreover, under NIR irradiation stimulation, osteocalcin, a late osteogenic differentiation marker, can be overexpressed by activating the Wnt signaling pathway. Mild heat stimulation activates the Wnt signaling pathway by up-regulating the expression of Wnt10b and A1p1 genes to stimulate bone regeneration [31]. 

**Figure 4:**
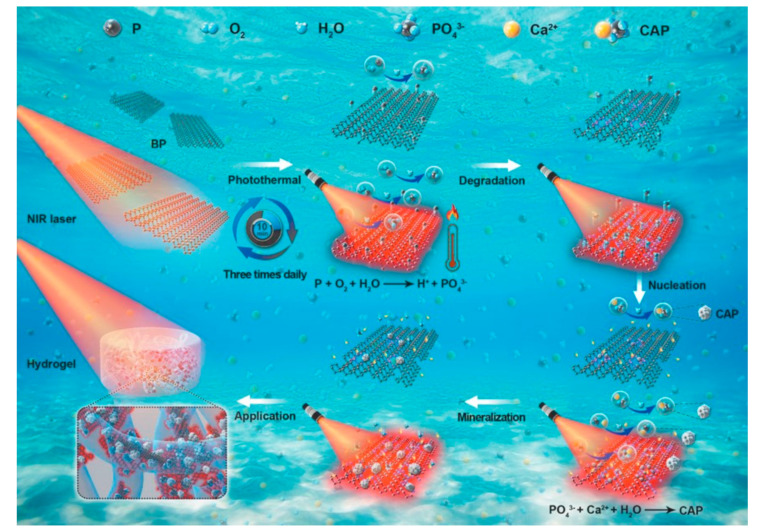
Mechanism of in situ mineralization of BP/agarose hydrogels under NIR irradiation [124]. Copyright 2020, Wiley.

BP, as an excellent photothermal conversion agent, can be oxidized to PO_4_^3−^ and form calcium phosphate (CAP) with calcium for in situ mineralization (Figure 4) [123,124]. CAP has excellent mechanical properties, which can be applied in bone defects of weight-bearing area [125]. Moreover, PO_4_^3−^ could promote osseointegration by being incorporated with Gd^3+^. The mechanism is as follows: (1) stimulate the M2 polarization of macrophages by releasing Gd^3+^; (2) promote angiogenesis by up-regulating VEGF in M2 polarized macrophages; and (3) achieve the deposition of calcification analogous to the Haversian system around the neovascularization [126]. 

**Figure 5:**
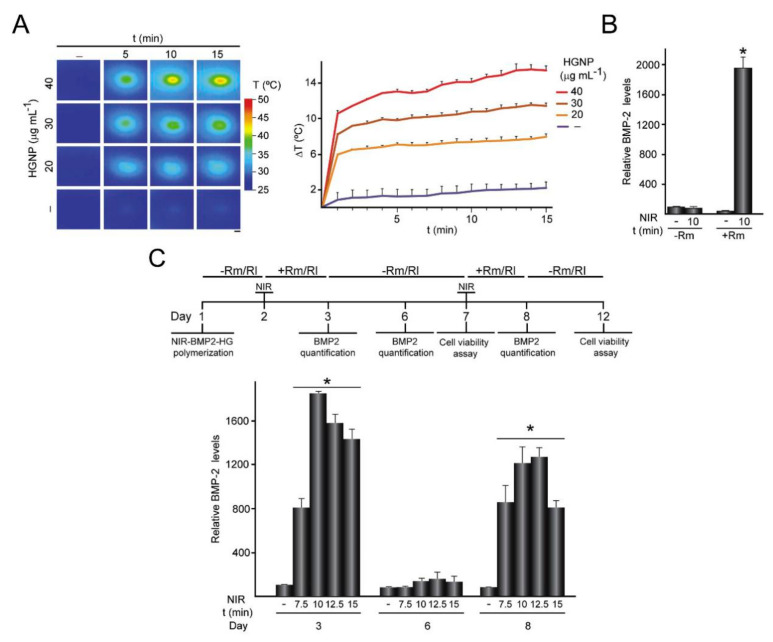
Activation of NIR-BMP-2-HG triggered by NIR light. (**A**) NIR-BMP-2-HG, polymerized with the indicated concentration of HGNP, were cultured for 1 day and then irradiated with NIR laser for the indicated times. (**B**) BMP-2 concentration in media conditioned by NIR-BMP-2-HG polymerized with 30 μg·mL^−1^ HGNP. (**C**) NIR-BMP-2-HG, polymerized with 30 μg·mL^−1^ HGNP, were NIR-irradiated in the presence of 10 nM Rm or 100 nM rapalog AP21967 (Rl). Timeline scheme of NIR-BMP-2-HG preparation, NIR irradiation of hydrogel (NIR), * *p* < 0.05, culture in the absence (-Rm/Rl) or presence (+Rm/Rl) of rapamycin or rapalog and analytical assays [127]. Copyright 2020, Elsevier.

Sanchez et al. fabricated a fibrin/GNPs hydrogel and C3H-BMP-2^high^ cells were successfully entrapped within the hydrogels (the composite hydrogel was named NIR-BMP2-HG). Under the dual stimulation of heat treatment induced by NIR irradiation (42–43 °C) and rapamycin (Rm), C3H-BMP-2^high^ cells can secrete BMP-2 to promote bone regeneration, and when heat treatment or rapamycin was used alone, BMP-2 secretion was negligible (Figure 5). BMP-2 is involved in the differentiation of BMSCs into osteoblasts and promotes the migration of BMSCs to the defect area [127,128]. The mild photothermal hydrogel increased the expression of heat shock proteins (HSP). Moreover, HSP70 can enhance the heat resistance of cells, and HSP47 promotes the maturation of osteogenic COL I. These two types of HSPs can promote cell osteogenic activity. Meanwhile, Ma et al. recently demonstrated that NIR irradiation + nHA could promote the expression of HSP47 and HSP70 [129].

## 4. Photothermal Hydrogels in Wound Healing and Bone Repair

For wound healing and bone repair, control of infection, cell proliferation, and angiogenesis are both key factors. The hydrogel can mimic the extracellular matrix in defect areas and provide a microenvironment for tissue regeneration. Further, the photothermal conversion ability of hybrid hydrogels play an important role in wound healing and bone repair. Mild heat (41 °C–43 °C) can promote cell proliferation, increase blood flow, and further induce tissue regeneration. Hyperthermia (>50 °C) can denature proteins, destroy the integrity of cell membranes, and inhibit the growth of bacteria. Therefore, photothermal hydrogels could be applied in various medical applications to treat infection and promote tissue regeneration. Table 1 summarizes the applications of hydrogels with PTT in wound healing and bone engineering.

### 4.1. Wound Healing

Common clinical traumas are caused by fractures, tumors, diabetes, etc., and are often accompanied by serious infections. To promote wound healing, many dressings have been developed to control infection, promote hemostasis, and improve angiogenesis [132,133,134]. The development of hydrogel with excellent antibacterial properties, good compatibility, and degradable ability is a promising strategy for wound healing [135,136]. Hydrogels with a photothermal conversion ability can regulate the temperature by changing the NIR irradiation intensity/time, photothermal initiator concentration/ratio, and cycling time [137,138]. Hyperthermia (>50 °C) can effectively inhibit bacterial growth, and mild heat (41 °C–43 °C) accelerates wound closure. For example, GelMA/BACA–Cu NPs hydrogels were prepared and possess good photothermal ability under laser exposure. After 10 min of NIR irradiation, the hyperthermia (>55 °C) of hydrogels can effectively inhibit the proliferation of bacteria, and Cu^2+^ played an antibacterial role with co-therapy of bacteria [139]. After 14 days, the wound closure rate of NIR+ GelMA/BACA–Cu NPs hydrogel group was 95.1%, while the control group showed a lower closure rate of about 79.3% [68]. Zhou et al. recently fabricated an oxidized dextran/PEG/CuS hydrogel and found that it played an important role in accelerating angiogenesis and promoting the proliferation of fibroblasts [140]. 

**Figure 6:**
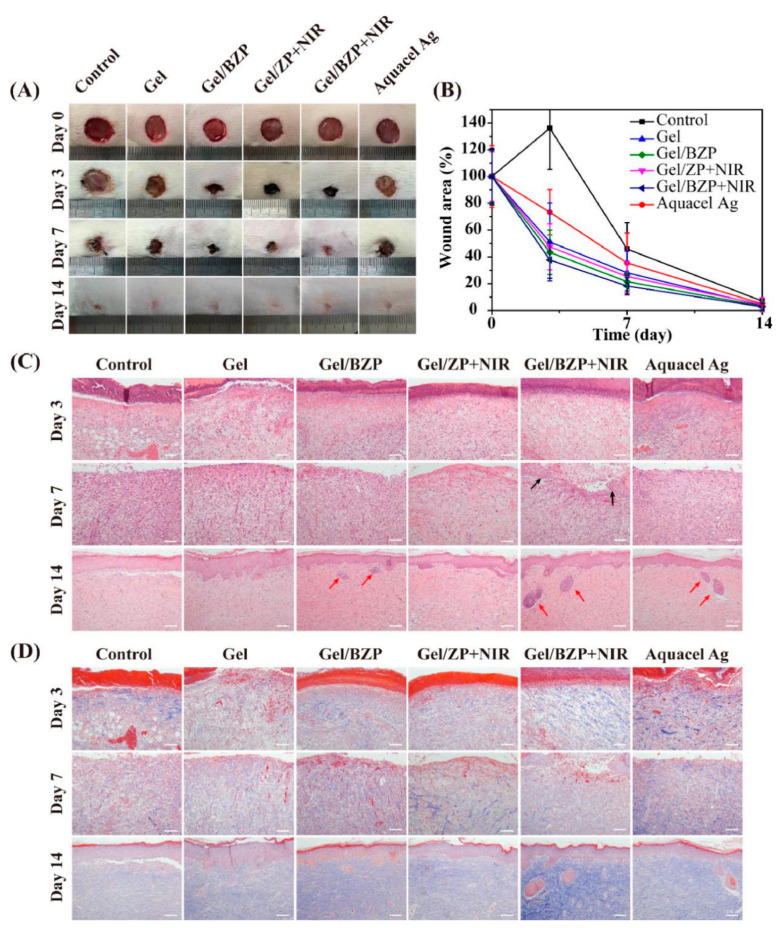
In vivo animal experiment assessment of hydrogels for wound healing. (**A**) Images of wounds treated with different hydrogels on 0, 3, 7, and 14 d. (**B**) Wound area values at different healing times. (**C**) H&E staining and (**D**) Masson’s trichrome staining of the wound section at 3, 7, and 14 d; scale bar = 100 μm. (ZP: ZIF8/PDA, BZP: BNN6/ZIF8/PDA) [121]. Copyright 2020, Elsevier.

Francisco et al. recently demonstrated that the CuS NP/fibrinogen hydrogel increased the capillary density compared to the control group, suggesting that Cu^2+^ can promote angiogenesis [141]. Huang et al. expounded that the GelMA/GO-βCD-BNN6 hydrogel could achieve the controlled release of NO under NIR irradiation. Hyperthermia (about 60 °C) and the NO released (4 µM) provided excellent antibacterial properties in vitro/vivo [82]. Moreover, the GelMA/oDex/BZP (BNN6/ZIF8/PDA) hydrogel showed NIR-irradiation dependent NO release. Figure 6 shows that the Gel+NIR group could accelerate wound healing. Masson’s trichrome staining and hematoxylin and eosin demonstrated that Gel+NIR group can effectively reduce tissue inflammation and accelerate the regeneration of skin appendages [121].

The effective control of inflammation is another key to wound healing. Sun et al. demonstrated that PEG/PDA hydrogel can effectively reduce the inflammatory response in methicillin-resistant *Staphylococcus aureus*-infected rats and accelerate wound healing [142]. Chu et al. recently developed a biomaterial with Cu-carbon dots + NIR and the wound closure rate reached 96% after treatments for 14 days, while the wound healing rate of the control group was 62%. Hematoxylin and eosin staining in the Cu-carbon dots + NIR group showed more neovascularization, collagen deposition, and re-epithelization than that in the control group [143].

Hyperthermia induced by NIR irradiation has a short-term antibacterial effect. When the NIR irradiation is stopped, the remaining bacteria cannot be effectively inhibited. To strengthen the antibacterial property of hydrogels, some studies have developed hydrogels loaded with antibacterial drugs for co-therapy [137]. Furthermore, NIR-triggered drug release had been successfully achieved. Ciprofloxacin (Cip) was loaded on PDA via π−π stacking and hydrogen bonds, which was released rapidly under NIR irradiation. The PDA NP-Cip+NIR group showed excellent inhibition of bacterial activity (2.1 × 10^6^ CFU/g) compared to the control group (6.0 × 10^6^ CFU/g) [131]. Deng et al. successfully fabricated a polysaccharide hydrogel embedded with ferric tannate (TA-Fe) nanoparticles and vancomycin. Under NIR irradiation, the vancomycin was released explosively. After the NIR was removed, a small amount of the vancomycin was released continuously. After 5 days of treatment, the Gel/vancomycin+NIR group showed the highest wound closure rate (80%) and erythema and edema were not observed [144].

Interestingly, the proliferation of bacteria often leads to a decrease in pH in the microenvironment. When the environment changes from an acidic to a neutral condition, the color of bromothymol blue (BTB), a pH-sensitive reagent, changed from yellow to green. Hence, Wang et al. designed a visual photothermal treatment strategy for infectious subcutaneous defects based on BTB. They employed PTDBD as a photo-initiator, β-glycerophosphate as a sol-gel conversion agent, and chitosan as scaffold material to obtain a visual antibacterial hydrogel. After 8 min of NIR irradiation, the temperature of the hydrogel up to 55 °C and showed a strong destructive effect on S. aureus [145,146].

### 4.2. Bone Regeneration

Bone defect repair usually requires the proliferation and differentiation of BMSCs, angiogenesis, and the deposition of calcifications. However, common clinical bone defect treatment, such as that for open fractures, are often accompanied by severe infection and tissue ischemic necrosis. As a reusable, nontoxic, and noninvasive new treatment strategy, local heat induced by PTT has shown excellent osteogenesis ability in vitro/vivo. The excellent biocompatibility, adjustable degradability, and porous structure of hydrogels can reduce the inflammatory response produced by implant materials and provide scaffolds for new bone-growth [147]. When hydrogels with PTT is applied to repair bone defects, it is divided into the following stages: (1) controlling infection to alleviate inflammation; (2) neovascularization to supply nutrients and oxygen; (3) promoting the proliferation and differentiation of BMSCs into osteoblasts to migrate toward the defect area; and (4) promoting calcification deposition and new bone formation.

Bone defects often require the implantation of artificial materials to promote bone regeneration, but irregular bone surfaces and implant materials may cause bacterial colonization and induce serious infections. Hyperthermia induced by PTT inhibits the growth of bacteria and may cause irreversible damage to normal cells [148]. Hence, researchers generally added antibacterial substances such as Cu^2+^ into the hydrogel to improve antibacterial properties. Heat (about 46 °C) induced by gold nanoparticles under NIR irradiation could stimulate BMP-2 expression in bone defect cells and promote the mineralization of new bone [149].

Recently research indicated that the incorporation of photothermal agents can enhance the osteogenic properties of hydrogels. BP/platelet-rich plasma (PRP)-chitosan hydrogel can effectively destroy diseased tissues such as rheumatoid arthritis through photothermal conversion and generate reactive oxygen species under NIR irradiation. PRP could improve the adhesion of BMSCs on the surface of the hydrogel. Then PO_4_^3−^ produced by degradation can form CaP by in situ biomineralization with Ca^2+^ to enhance the osteogenesis process [130]. Luo et al. recently demonstrated that an oxidized sodium alginate/CS/PDA/nHA hydrogel showed high osteogenic ability because of the adhesion ability of PDA. In the long-term bone regeneration process, osteoblasts gradually proliferated, differentiated, and migrated to the defect area [150].

Previous research demonstrated that a single NIR irradiation has no significant effect on osteoblast proliferation. Periodic and long-term local mild heat stimulation can upregulate the expression of osteoblast-related genes. Xue et al. recently found that with a BGN/PDA/fibrin glue hydrogel and MC3T3-E1 cells co-cultured for 3 days with 10 min of NIR irradiation, there was negligible difference in MC3T3-E1 cells with and without NIR irradiation [151]. Compared to a single NIR irradiation, Ma et al. demonstrated that nHA/GO/CS scaffolds cultured with MC3T3-E1 cells (42 ± 0.5 °C for 60 s for 3 d) could significantly promote cell proliferation [129]. Zhang et al. also found a significant increase in cell proliferation on HA/graphene/CS scaffolds cultured with MC3T3-E1 cells (under NIR irradiation for 3 min to 43 °C for 3 days) [152]. This evidence suggests the necessity of periodic thermal stimulation in promoting osteogenesis. 

**Figure 7:**
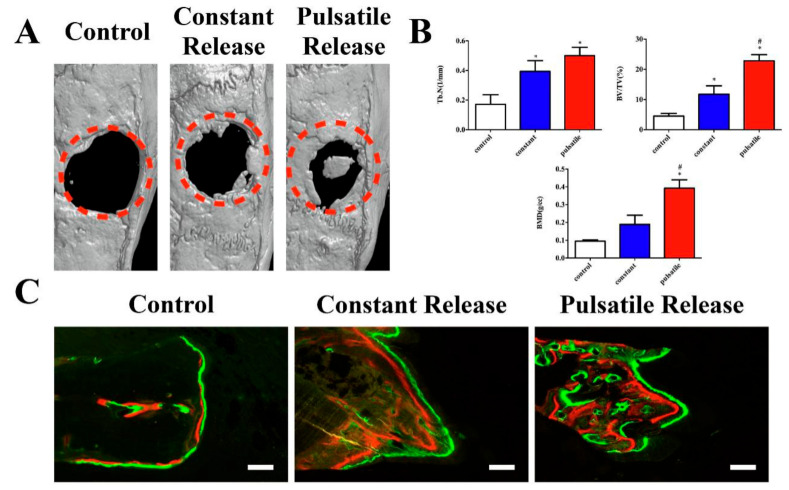
Micro-CT scanning and sequential fluorescent labeling. (**A**) Representative 3D reconstruction of the 4 mm calvarial defect from each group; (**B**) quantitative comparison of Tb.N, BV/TV and BMD among the control, constant release and pulsatile release groups. (**C**) Sequential fluorescent labeling of alizarin red (red) and calcein (green) represented the bone formation at 3 and 6 weeks. * *p* < 0.05 compared to the control group, # *p* < 0.05 compared to the constant release group. Scale bar = 20 μm [153]. Copyright 2021, Elsevier.

To bone regeneration, the microenvironment regulated by signal molecules, such as parathyroid hormone (PTH), is crucial. However, unpredictable drug distribution and suboptimal local concentration limit its therapeutic effect. Wang et al. recently fabricated a CS/rGO hydrogel film and successfully loaded with teriparatide (PTH 1–34, an FDA-approved drug for osteoporosis treatment), which achieved pulsed release under NIR irradiation. In the osteoporotic rat model of bone defect, the local pulsatile teriparatide release group showed a higher ratio of new bone formation (BV/TV: 22.80%) than the control group (BV/TV 4.51%) (Figure 7) [153].

Moreover, some studies have reported a new hemicyanine dye LET-3 that can promote osteogenesis and realize photoacoustic imaging. LET-3 can be anchored on a calcium silicate scaffold, and further improve the expression of ALP. Especially, under NIR irradiation, higher ALP activity could induce a stronger photoacoustic effect. Hence, it is worth exploring to realize the noninvasive dynamic monitoring of osteogenesis in vivo through photoacoustic effect [154]. From this, researchers can design hydrogels with photothermal effects to promote osteogenesis and observe the process of osteogenesis using noninvasive imaging. 

## 5. Prospective and Conclusion

Hydrogels with photothermal effects have gained increasing attention and are applied widely in wound healing and bone regeneration. It is one of the research hotspots in recent years to take advantage of the NIR absorption properties to achieve the target temperature. Besides, hyperthermia can be used for antibacterial and antitumor therapy, as mild local heat can simulate the hot spring effect to promote cell proliferation and accelerate wound healing. NIR-induced fluorescence imaging can also be used to track the development of bone regeneration dynamically and noninvasively. Remarkably, high temperature generated by photothermal materials under NIR irradiation, self-disinfecting personal protective equipment, as face masks, can be developed to fight COVID-19. This may be a promising strategy for the development of photothermal materials.

However, the low tissue penetration rate of NIR limits the application of PTT in deep tissue diseases. Besides, some materials have both photothermal conversion properties and magnetothermal conversion ability. To control drug release, photothermal-trigger release, pH-trigger release, and magnetothermal-trigger release are the key directions for future development. 

In conclusion, hydrogels with PTT have excellent antibacterial effects and can promote tissue repair. In the future, researchers can make full use of the advantages of PTT to incorporate it in other treatment methods to overcome shortcomings, broaden applications, improve curative effects, and reduce costs. These advantages of PTT and hydrogels provide a new strategy in wound healing and bone tissue engineering.

## Figures and Tables

**Table 1:** Hydrogels with PTT for wound healing and bone engineering.

Hydrogels	Photothermal Agents	Concentration	Intensity and Time	Temperature	Application	Reference
NOCS/OSA/FA	FA(Fe_2_SiO_4_)	5 mg/mL	0.36 W/cm^2^ 10 min	40 °C	Ion release Angiogenesis	[112]
CMCS/OSA/ CuS	CuS	0.8 mg/mL	1 W/cm^2^ 5 min	50 °C	Antibacterial Angiogenesis	[103]
CuS/HA	CuS	0.2 mg/mL	1 W/cm^2^ 10 min	53.1 °C	Wound healing Angiogenesis	[115]
NIPAAm/ AAm/CuS/ mSiO2	CuS/mSiO_2_ NPs	1.5 mg/mL	2 W/cm^2^ 8 min	59.5 °C	Antibacterial effect	[113]
GelMA/BP	BP	1 mg/mL	1 W/cm^2^ 5 min	55.3 °C	Antibacterial effect Bone regeneration	[125]
BP/CS/PRP	BP	0.05 mg/mL	1 W/cm^2^ 8 min	45 °C	Drug release Antiarthritic	[130]
PDA/GC/Cip	PDA NPs	4 mg/mL	0.5 W/cm^2^ 10 min	46.8 °C	Antibacterial effect Drug release	[131]
Gel-PDA/Cur	PDA/Cur	2 mg/mL	1 W/cm^2^ 10 min	50.9 °C	Antibacterial effect Drug release	[95]
MPDA/GO/ CNF	MPDA/GO	10 mg/mL	2 W/cm^2^ 10 min	56 °C	Drug release	[90]
GelMA/oDex/BNN6@ZIF8/ PDA	PDA/ZIF8	1 mg/mL	2 W/cm^2^ 10 min	50 °C	Antibacterial effect Angiogenesis	[121]

## Data Availability

The data presented in this study are available on request from the corresponding author.
